# A formalin-free method for stabilizing cells for nucleic acid amplification, hybridization and next-generation sequencing

**DOI:** 10.1186/s13104-015-1725-4

**Published:** 2015-12-09

**Authors:** Jianbing Qin, Jennifer N. Sanmann, Jeff S. Kittrell, Pamela A. Althof, Erin E. Kaspar, Bradford A. Hunsley

**Affiliations:** Research and Development Division, Streck, Inc., 109th Street, Omaha, NE 68128 USA; Munroe-Meyer Institute for Genetics and Rehabilitation, Cytogenetic and Human Genetics Laboratories, University of Nebraska Medical Center, Omaha, NE 68198 USA; Genetics, Cell Biology and Anatomy, University of Nebraska Medical Center, Omaha, NE 68198 USA

**Keywords:** Formalin-free fixative, Cell fixation, Nucleic acid amplification, FISH, NGS

## Abstract

**Background:**

Formalin has been widely used by pathology laboratories. Its carcinogenicity has led researchers to explore formalin substitutes. Streck Cell Preservative (SCP) is a formalin-free preservative that can preserve cellular antigens. This study was undertaken to investigate the effects of cell preservation using SCP on nucleic acid amplification, hybridization, and next-generation sequencing (NGS) as compared to control frozen cells and cells fixed in the traditional cell and tissue fixative, 10 % neutral buffered formalin (NBF).

**Findings:**

The breast cancer cell line, SKBR-3, was used as a model system. Prior to nucleic acid extraction and fluorescence in situ hybridization (FISH), cells were fixed in SCP or NBF overnight at room temperature with frozen cells in parallel. Analysis showed that similar DNA extraction yields and amplification profiles determined by PCR in SCP preserved cells and control frozen cells, whereas NBF preserved cells had decreased DNA yield and impaired PCR amplification. Molecular cytogenetic studies by FISH technique indicated that the ratios of *ERBB2* (HER-2/neu) signals to the chromosome 17 centromere (CEP17) were comparable for frozen cells and SCP preserved cells. The fluorescence images of both SCP fixed and control frozen cells were also clear and comparable. On the contrary, the same analysis was unsuccessful with NBF preserved cells due to poor hybridization quality. Our data also demonstrated that SCP had negligible effect on NGS testing.

**Conclusion:**

We conclude that SCP can be used as an alternative to NBF as a preservative for maintaining the integrity of nucleic acids for nucleic acid amplification, sequencing and FISH analysis.

**Electronic supplementary material:**

The online version of this article (doi:10.1186/s13104-015-1725-4) contains supplementary material, which is available to authorized users.

## Background

Preservation of cell and tissue morphology and antigenicity in clinical specimens is the foundation step in accurate diagnoses. Formalin has been a traditional and popular choice of preservative for this purpose [1]. Formalin preserved cells and tissues exhibit excellent morphology and acceptable antigenicity, but there are some major drawbacks. Studies show that formalin exerts a carcinogenic effect and poses health risks to the laboratory users [2]. Formalin causes detrimental effects on biomolecules, mainly by crosslinking reactions [3, 4]. The later effect is particularly relevant in nucleic acid based molecular diagnosis, as nucleic acids are often fragmented, chemically modified and difficult to extract from a formalin preserved pathological specimen [4–6]. During the last two decades, many attempts have been made to optimize existing processing procedures or introduce alternative fixatives to formalin, but formalin is still widely used as the preferred cell and tissue preservative in pathology laboratories [7–9].

Streck cell preservative (SCP) is a formalin-free preservative [10]. Previous studies demonstrate that SCP preserves cellular antigens for immunophenotyping by flow cytometry [11]. In this study, we compared nucleic acid integrity in samples preserved using SCP and formalin. We utilized droplet digital PCR (ddPCR) based technique to study nucleic acid amplifiability and fluorescence in situ hybridization (FISH) analysis for cytogenetic integrity. Compatibility of SCP with next-generation sequencing (NGS) technique was evaluated as well, as these methods are in the frontier of present day molecular diagnostic field due to their high sensitivity and versatility [12–14].

## Methods

### Chemicals and materials

Formaldehyde (Cat. No. BDH0500-1LP) was purchased from BDH (Epping, NSW, Australia) and NaH_2_PO_4_ & Na_2_HPO_4_ from Sigma-Aldrich (St. Louis, MO). Streck Cell Preservative™ (SCP) was obtained from Streck Inc., Omaha, NE, USA. Colcemid^®^ solution (Cat. No. 9311) was purchased from Irvine Scientific (Irvine, CA, USA). Glacial acetic acid (Cat. No. 2504-14) was purchased from Mallinckrodt Chemical Inc. (St. Louis, MO, USA). Anhydrous methanol (Cat. No. AC61009-0040) was purchased from Acros Organics (Geel, Belgium).

### Cell preservation for DNA experiments

The breast cancer cell line, SKBR-3, was obtained from American Type Culture Collection (Rockville, MD, USA) and routinely passaged in ATCC formulated modified McCoy’s 5a medium (Cat. No. 30-2007) containing 10 % fetal bovine serum at 37 °C in humidified atmosphere of 5 % CO_2_.

SKBR-3 cells grown to 70–80 % confluence were tripsinized and washed two times with phosphate buffer solution (PBS) and suspended in fresh PBS. The cells were equally divided into three sets and transferred into three eppendorf tubes containing 1 × 10^6^ SKBR-3 cells. One set of cells was snap frozen at −80 °C to be used as a control. The cells in the second tube were suspended in 1 mL SCP diluted with PBS in 1:1 volume ratio. The third set of cells was suspended in 1 mL of 10 % neutral buffered formalin (NBF). 10 % NBF was prepared according to the standard protocol (100 mL of formaldehyde, 900 mL of distilled water, 4.0 g of NaH_2_PO_4_, 6.5 g of Na_2_HPO_4_, mix to dissolve.). Both tubes were incubated at room temperature overnight (15–18 h). At the end of the incubation period, the cells were washed twice with PBS and cell pellets were used to extract DNA.

### DNA extraction

DNA was extracted from control cells (snap frozen) as well as cells fixed with 10 % NBF and SCP using QIAamp DNA FFPE Tissue kit (Qiagen, Santa Clarita, CA). The manufacturer’s recommended protocol was followed with slight modification. Steps 1–9 of the protocol were omitted. DNA extraction began at step 10 by treating the cell pellet (1 × 10^6^ cells) with buffer ATL and proteinase K. Steps 11–21 in the manufacturer’s recommended protocol were followed. Finally, DNA was eluted in 60 µL of elution buffer (Buffer ATE). DNA concentration was determined using Qubit^®^ 2.0 fluorometer and Qubit^®^ dsDNA HS assay kit (Invitrogen, Eugene, Oregon, USA). DNA was stored at −80 °C until analysis by ddPCR or NGS.

### DNA analysis by ddPCR

β-actin copy number assay: Additional file 1: Table S1 provides sequence information for primers and probes for the ddPCR assay to amplify long, medium and short amplicons from the β-actin gene. All primers were purchased from Integrated DNA Technologies (IDT) (Coralville, IA). A PCR master mix, 2X ddPCR™ Supermix for Probes, was purchased from Bio-Rad Laboratories (Hercules, CA). Final concentrations of primers and probe in PCR reactions were 900 nM and 250 nM, respectively, in a final volume of 20 μL. Equal amount of DNA (5 ng) from each sample was used for PCR reactions. A Bio-Rad QX100 Droplet Digital™ PCR System was used as described by Hindson and colleagues [12]. Thermal cycling was performed with a Bio-Rad C1000 Touch Thermal Cycler. The following PCR conditions were used: 10 min at 98 °C, 40 cycles of 30 s at 95 °C, 30 s at 54 °C, and 30 s at 72 °C. A final extension was done for 10 min at 72 °C followed by a heating step for 10 min at 98 °C to inactivate the polymerase. Data analysis was performed using Bio-Rad QuantaSoft software version 1.3.2.

HER-2/CEP17 ratio assay: *ERBB2* (HER-2/neu) status is defined as the ratio between *ERBB2* (HER-2/neu) copy number and chromosome 17 centromere copy number (CEP17). For the detection of HER-2 DNA copy number, *ERBB2* assay (HER-2: Hs02803918_cn, Applied Biosystems) was used. As previously described [15], primers and probe for CEP17 assay were synthesized and assays were carried out using the ddPCR system.

### DNA analysis by NGS

Genomic DNA libraries were prepared from snap frozen and SCP derived DNA using Illumina^®^ TruSight Rapid Capture kit (Cat. No. FC-140-1101). According to the manufacturer’s protocol, 10 μL of DNA at 5 ng/10 μL (n = 3) was used in the tagmentation reaction, followed by limited-cycle PCR amplification. After clean up, the DNA libraries were pooled and hybridized with capture probes using Illumina TruSight Cancer sequencing panel (Cat. No. FC-121-0202). To ensure high specificity of the captured regions, a second hybridization and capture process was performed. The captured libraries were finally amplified via another limited-cycle PCR program and sequenced on an Illumina HiSeq 2500 sequence analyzer. The reads were pre-processed using fqtrim (http://ccb.jhu.edu/software/fqtrim/index.shtml) to remove any N that was present. The resulting reads were sequentially aligned to hg19 human genome (UCSC version, February 2009) using the Burrows-Wheeler alignment tool.

### *ERBB2* (HER-2/neu) FISH assay

SKBR-3 cells were grown in three T-25 flasks to approximately 75 % confluence. A 50 µL solution (10 µg/mL) of Colcemid was added to each flask and incubated for 20 min at 37 °C. Cells were removed from each flask by trypsinization, transferred to a 15 mL tube and pelleted by centrifuging at 1000 rpm for 8 min. The cell pellets were re-suspended in 6 mL of hypotonic solution (0.075 M KCl warmed to 37 °C) and incubated for 20 min at 37 °C. For the control sample, 1 mL of 3:1 methanol:glacial acetic acid fixative was added to the cells and incubated at room temperature for 5 min before centrifugation at 1200 rpm for 6 min to remove hypotonic/fixative supernatant. Cells were washed three times with 6 mL of the same fixative and finally re-suspended in 1.5 mL of the fixative for storage at 4 °C. For the SCP and NBF test samples, cells were first incubated with hypotonic solution, washed with PBS and then suspended either in 1 mL of SCP:PBS (1:1) or in 10 % NBF. After incubation at room temperature for 18 h, preserved cells were washed and re-suspended in PBS for storage at 4 °C.

Slides were prepared for fluorescence in situ hybridization (FISH) at 25 °C and 40 % relative humidity using a cytogenetic drying chamber (Thermatron, Holland, MI) and were aged at 100 °C for 2 min. Interphase FISH studies were performed using the PathVysion HER-2 DNA Probe Kit (Abbott Molecular, Des Plaines, IL) specific for the *ERBB2* (HER-2/neu) locus at 17q11.2-q12 and the 17 centromere (D17Z1). The cells and probes were co-denaturated at 78 °C for 3 min using the ThermoBrite™ system (Abbott Molecular) and incubated overnight at 37 °C in a humidified chamber.

Nuclei were counterstained with 4, 6-diamidino-2-phenylindole (DAPI) in Antifade solution (Abbott Molecular), and the slides were analyzed using a Leica DM6000B fluorescence microscope (Leica Biosystems, Inc., Buffalo Grove, IL). Hybridization signals for *ERBB2* (HER-2/neu) and the 17 centromere were assessed in 30 interphase nuclei per specimen, and the observed signal patterns were interpreted utilizing the 2013 ASCO/CAP *ERBB2* (HER-2/neu) reporting guidelines [14]. Images were acquired and archived using the CytoVision Image Analysis System (Leica Biosystems, Inc.).

### Statistical analysis

Statistical analysis was carried out using Microsoft Excel for Office 2007. Analysis was performed using paired, two-tailed Student’s *t* test and p < 0.05 was considered statistically significant.

## Findings

### Effect of fixatives on DNA yield and PCR amplification

Cultured SKBR-3 cells were re-suspended in SCP (1:1 diluted with PBS) or 10 % NBF and stored overnight at room temperature with unfixed frozen cells in parallel. Genomic DNA was purified from these three different groups of cells and quantified by the fluorescence-based Qubit^®^ assay. The mean DNA yield from unfixed control (frozen) cells was 2.4 ± 0.7 µg/10^6^ cells. Similar amounts of DNA were obtained from SCP preserved cells with a mean concentration of 2.2 ± 0.7 µg/10^6^ cells, showing no statistically significant difference from control cells (*p* = 0.116, n = 10). Conversely, fixation of cells by 10 % NBF resulted in a significantly lower (*p* = 0.0001) DNA yield than the control or SCP samples. The mean DNA yield of NBF fixed cells was 0.14 ± 0.2 µg/10^6^ cells.

**Fig. 1:**
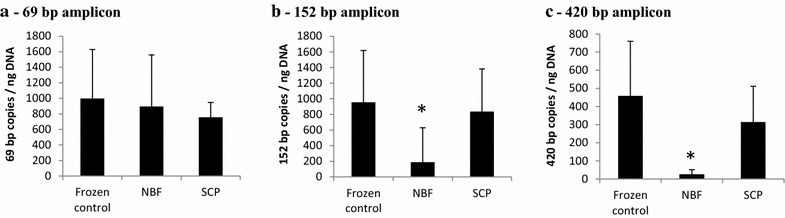
DNA integrity assayed by β-actin ddPCR. DNA templates were extracted from snap frozen SKBR-3 cells and cells treated with 10 % NBF or SCP. Three different fragment lengths from the same target gene β-actin were amplified. The sizes of three nested amplicons are 69 bp (**a**), 152 bp (**b**) and 420 bp (**c**), respectively. Mean copy numbers are shown with *error bars* indicating standard deviation, n = 10. *P < 0.005

Potential DNA fragmentation and damage caused by fixation was assessed using two different PCR amplification assays. The first assay was designed to amplify a series of fragment lengths from the same target gene, β-actin. The sizes of three nested amplicons are 69 base pairs (bp), 152 and 420 bp, respectively. As shown in the Fig. 1a, the average copy numbers of the 69 bp β-actin amplicon for the control, SCP and NBF treated cells were 996, 756 and 896 copies per ng DNA, respectively (n = 10). In contrast, for the amplification of 152 bp amplicon (Fig. 1b), the mean copy numbers were 955, 836 and 188 copies per ng DNA for control, SCP and NBF treated samples, respectively. A similar pattern of the amplifiable input DNA was observed in the 420 bp fragment test (Fig. 1c) as the 152 bp amplification. The detected copy number was decreased dramatically from 459 copies per ng DNA of control cells or 314 copies per ng DNA of treated cells to 25 copies per ng DNA of NBF fixed cells. For all these tests, there was no statistically significant difference in the amount of amplifiable DNA between frozen control and SCP treated cells. However, cells treated with NBF showed statistically significant decrease in target copy numbers of 152 bp and 420 bp compared to the control cells.

In another ddPCR assay, HER-2 genomic DNA copy number alterations were measured and normalized to the CEP17 reference gene. Table 1 shows HER-2 and CEP17 genomic DNA copy numbers and HER-2 to CEP17 ratios (n = 10). NBF fixation impaired PCR amplification resulting in significant reduction in HER-2 and CEP17 copy numbers. As compared to the control samples, cells treated with NBF showed 63.0-fold and 397.6-fold decreases in HER-2 and CEP17 copy numbers, respectively. This dramatic decrease in CEP17 copy number contributed to the abnormally high HER-2/CEP17 ratio (44.3) in NBF treated cells. In contrast, SCP treatment slightly affected amplifiable DNA available for the PCR assay. There was about 1.4-fold decrease in both HER-2 and CEP17 copy numbers. The HER-2/CEP17 ratios were similar for the control (7.0) and SCP treated (6.9) cells (Table 1).

**Table 1:** HER-2/CEP17 ratio in control and fixed SKBR-3 cells as determined by ddPCR (mean ± SD)

Sample Type	HER-2	CEP17	HER-2/CEP17
	Copies/10^6^ cells	Copies/10^6^ cells	Ratio
Frozen control cells	10,288,417 ± 4,535,976	1,465,957 ± 763,934	7.0 ± 1.1
10 % NBF fixed cells	163,256 ± 147,676	3,687 ± 3,815	44.3 ± 12.8
SCP fixed cells	7,380,677 ± 4,069,048	1,063,169 ± 678,786	6.9 ± 1.4

### Effect of fixatives on DNA hybridization

**Fig. 2:**
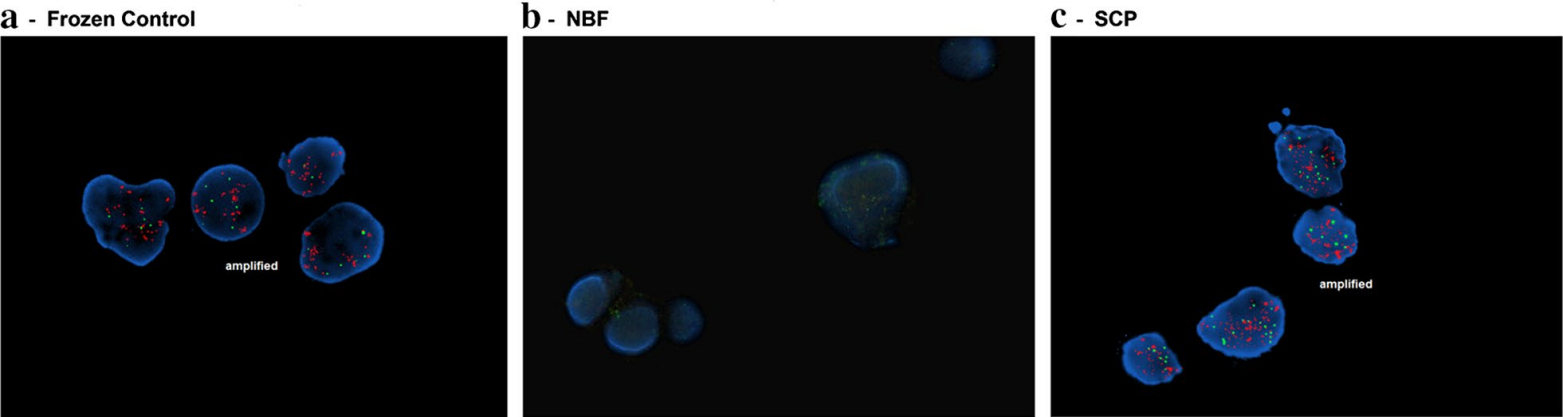
DNA hybridization assayed by ERBB2 (HER-2/neu) FISH. SKBR-3 cells were snap frozen (**a**) or exposed to 10 % NBF (**b**) or SCP (**c**) prior to FISH analysis. Representative cell nuclei are shown for HER-2 amplification (*green*) and multiple copies of CEP17 (*red*), as well as countstain with DAPI (*blue*). n = 5

FISH studies were performed for assessment of the *ERBB2* (HER-2/neu) locus and the chromosome 17 centromere for controls cells and those cells treated with SCP or NBF. As shown in Fig. 2, hybridization of HER-2 and the chromosome 17 centromere was unsuccessful for the cells exposed to NBF in all experiments (n = 5). Conversely, no hybridization failure was observed in any of the control or SCP cell preparations performed simultaneously. Qualitatively, the cells exposed to SCP exhibited crisper, more precise chromosome 17 centromere signals as compared to the control cells. The HER-2 signals in the SCP exposed cells were slightly dimmer than the HER-2 signals when compared to the control cells; however, the signal intensity was not prohibitive for assessment or enumeration of the HER-2 locus in either the SCP exposed or control cells.

The mean copy number of HER-2 and chromosome 17 centromere signals in the SCP cells was 52.84 and 6.40, respectively; there was no statistically significant difference from control cells for either of these loci (p = 0.23 and p = 0.27, respectively). The mean ratio of HER-2 to the chromosome 17 centromere for SCP cells was 8.27 and was also not statistically significant from the mean ratio of HER-2 to 17 centromere in the control cells (p = 0.39). Given the lack of successful hybridization for the HER-2 and 17 centromere loci in the NBF preserved cells, comparison with this population was not possible.

### NGS application of DNA from SCP treated samples

To explore if SCP-derived DNA is compatible with NGS technology, we did an Illumina-based sequencing study. As described in “Materials and Methods” section, sequencing libraries of DNA extracted from snap frozen or SCP-treated SKBR-3 cells were prepared using Illumina TruSight Rapid Capture kit and capture enriched by TruSight Cancer Sequencing Panel. An Illumina HiSeq 2500 sequence analyzer was utilized as the NGS platform in this study. We first compared common NGS quality parameters across the two types of samples, i.e., snap frozen and SCP treated (Table 2). There was no statistically significant difference in mapping results obtained from two sample types. The percentage of bases that were mapped was 51.3 and 50.8 % for frozen and SCP samples, respectively. The estimation of error rates indicated by discrepancies also showed no significant difference between the two sample types. Similar numbers of base with Phred mapping score of 20 or less per reads were also observed in the paired frozen and SCP samples.

**Table 2:** Comparison of average sequencing quality metrics

	Frozen control	SCP	p value
Total reads (×10^6^)	24.6 ± 0.33	34.1 ± 1.86	0.01
Unique reads (×10^6^)	2.89 ± 1.26	4.75 ± 0.63	0.23
Mapped unique reads (×10^6^)	2.45 ± 1.11	4.15 ± 0.60	0.22
Bases on-target^a^ (×10^6^)	89.8 ± 47.4	177 ± 32.16	0.19
% Bases on-target	51.3 ± 0.02	50.8 ± 0.00	0.69
Total discrepancies^b^ (×10^5^)	1.17 ± 0.31	1.75 ± 0.27	0.14
Discrepancies per read	0.06 ± 0.04	0.04 ± 0.00	0.55
Number of bases <Q20^c^ (×10^6^)	0.77 ± 0.29	1.23 ± 0.26	0.20
Number of bases <Q20 per reads	0.46 ± 0.20	0.60 ± 0.61	0.63
Mean insert size	98 ± 5.51	119 ± 5.20	0.07

^a^On-target means mapping to the targets

^b^Discrepancies mean any base called in a read that is different from the

reference sequence

^c^Q20: Phred mapping quality score of 20

We further examined if the SCP treatment affected sequencing of target regions of interest. The TruSight Cancer Sequencing Panel utilized in this study covers 94 target genes. As summarized in Additional file 2: Table S2, there were only minor differences in average depth of coverage by gene between frozen and SCP samples and none of these differences were statistically significant.

## Discussion

Significant advancement has been made in recent years in molecular testing technologies. As such, molecular diagnostic tests are being developed to complement traditional histological and immunohistological diagnostic tests in disease diagnosis [16]. Although formalin has been widely used by pathology laboratories to preserve histomorphologic features of specimens, its carcinogenicity and adversity towards molecular integrity have prompted researchers to explore formalin substitutes. SCP is a non-formalin fixative designed to preserve cellular antigens. However, applications of SCP to samples used in molecular testing were yet to be assessed. This study was designed to investigate effects of this fixative on cellular nucleic acid extraction yield and integrity as compared to 10 % NBF and snap frozen samples using the SKBR-3 cell line as a model.

Formalin can interact with macromolecules in multiple ways. For example, formalin fixation induces not only protein–protein, but also DNA–protein cross-linking [4, 16]. The protein networks resulting from protein–protein cross-linking may cause DNA trapping, whereas DNA–protein cross-linking reduces DNA solubility. These effects make it difficult to extract DNA from NBF fixed samples. In addition, formalin can also cause DNA degradation via hydrolysis of phosphodiester bonds [4]. The combination of these factors may explain why the DNA yield recovered from NBF treated cells was significantly lower than the yield from snap frozen control cells. Similarly, Serth et al. reported that formalin fixation strongly impaired DNA yield [17]. Heating can reverse some of cross-linking effects of formalin [18]. Both Serth’s and our DNA isolation protocols contained heating steps, but cross-linking caused by formalin can still be problematic after heating if extensive cross-linking occurs. Moreover, spectrophotometry is not suitable for quantification of DNA derived from a fixed sample [19]. Thus, we used a fluorescence-based method to measure double stranded DNA concentration. Another advantage using this method was to avoid interference of RNA contaminated in DNA samples. However, chemical modification to DNA functional groups can inhibit DNA double helix formation, resulting in decreased fluorescence intensity and low DNA quantity [20]. Interestingly, non-formalin SCP fixed samples yielded a similar amount of DNA as snap frozen samples. This indicates a chemically unaltered DNA structure in SCP preserved cells.

PCR is a powerful tool to measure the fraction of DNA that can be amplified, an indicator of DNA quality/integrity and the size distribution of fragmented DNA. In the present study, nested fragments of three different lengths from the same target DNA were amplified in the β-actin ddPCR assay. On the one hand, the target copy numbers of NBF samples were significantly lower than those in control or SCP samples in both the large amplicon size test of 420 bp and the medium amplicon size test of 152 bp, but not in the short ampilcon test of 69 bp. This was largely due to the DNA fragmentation effect caused by NBF fixation. The fragmentation significantly reduced the amplifiable DNA input for relatively long PCR amplification tests. On the other hand, PCR efficiency can be affected by amplicon size. Large amplicons leads to decreased PCR efficiency. Target copy numbers moderately decreased from 996 of 69 or 955 of 152 to 459 of 420 bp for the snap frozen control samples. The SCP samples showed a similar pattern as control cells. However, a dramatic decrease in target copy numbers was observed in NBF samples. For example, the copy number was reduced 36 fold from 896 of 69 to 25 of 420 bp for NBF samples. The changes in the frozen control and SCP samples were only 2.2 and 2.4 fold, respectively. Taken together, these moderate fold changes found in the control or SCP samples were likely due to the PCR efficiency effect, whereas the much larger fold change observed in the NBF samples was a result of the combined effects of fragmentation and PCR efficiency, with the former as the main driving effect. This suggests remarkable fragmentation of DNA in NBF fixed samples, but not in SCP or snap frozen control samples.

HER-2 is a member of the human epidermal growth factor receptor family. Amplication or overexpression of HER-2 is involved in development and progression of certain types of cancer, particularly breast cancer. Thus, determination of HER-2 status is extremely useful in the assessment of breast cancer for treatment purposes [13]. The SKBR-3 model used in the present study is a breast cancer cell line with known alterations in HER-2 and CEP17. We examined the effect of fixatives on HER-2 copy number measurements by ddPCR. Similar to the β-actin ddPCR result, SCP treatment only caused minimal decreases in both HER-2 and CEP17 copy numbers compared to the control sample. The HER-2/CEP17 ratios of control and SCP samples were similar to each other, suggesting SCP’s effect on HER-2/CEP17 testing was negligible. However, NBF fixation resulted in a very low HER-2 copy number. Moreover, CEP17 copy number was even further diminished. One possibility is that CEP17 DNA region is more prone to fragmentation from NBF fixation. Because of the remarkable decrease in CEP17 copy number, the HER-2/CEP17 ratio was 6.3 times higher in NBF than in control or SCP samples. FISH is another way to assess HER-2 amplification, which is routinely performed in current clinical settings. Our FISH data matched PCR data in regard to the effect of fixatives on HER-2 amplification testing. In particular, the mean HER-2/CEP17 ratio of SCP cells from FISH was 8.27. This is comparable to the ratios of 7.0 and 6.9 obtained from the PCR assay for control and SCP cells, respectively. Our data is also confirmed by Pat et al. who reported a HER-2/CEP17 ratio of 7.25 in SKBR-3 cells using FISH analysis [21]. The hybridization failure observed in NBF cells may be due to poor probe penetration through fixed cell membrane, DNA fragmentation or both.

NGS has emerged as another powerful molecular technique. We observed some rare discrepancies between snap frozen and SCP cells. The total number of reads generated from frozen samples was lower than that from SCP samples. This is likely explained by slight DNA degradation of frozen samples during storage. It can also be the possible reason why the frozen samples produced a slightly smaller mean insert size than SCP samples. However, our data revealed that SCP had little effect on common sequencing quality metrics and average depth of coverage for individual genes. These findings suggest that SCP preservation posed no or negligible effects on NGS testing, and DNA derived from SCP treated samples is suitable for NGS analysis.

As with DNA, it is also well known that formalin can react with RNA causing damage, fragmentation and altered amplification profiles [22]. We did a similar study of small sample size to compare the effects of fixatives on RNA performance. Our preliminary data showed that SCP was compatible with RNA extraction and reverse transcription PCR amplification of HER-2 mRNA and GAPDH mRNA, whereas NBF fixation resulted in a very poor RNA yield and significantly low mRNA copy numbers (data not shown).

Utilization of molecular testing is growing. It is essential to develop formalin substitute fixatives for molecular assay development and clinical practice without significantly compromising histomorphologic features. A number of molecular fixatives have been reported. For example, alcohol-based fixatives such as CyMol, UPM, and UMFIX can penetrate into and fix tissues quickly by denaturing, instead of cross-linking, proteins [9, 23]. However, tissues can become very hard and cells are prone to shrinkage during alcohol-based fixative process [24]. SCP contains no alcohol or formalin. To our knowledge, this is the first study focusing on SCP molecular preservation features. Our research demonstrated that SCP had little adverse effects on widely used molecular technologies. Although using a cancer cell line model is a good choice for a pilot investigation like this study, it would be very interesting to perform similar studies on tissues samples, especially surgical biospecimens. In the future, we hope to investigate various preanalytical factors of SCP fixation such as fixation time, specimen size, and type of tissue. Collecting more data on RNA and NGS testing will be beneficial too.

In conclusion, our study provides experimental evidence that cells preserved with SCP can maintain the integrity of nucleic acids for downstream molecular analysis including PCR amplification, FISH, and NGS. SCP provides key advantages in nucleic acid detection and can be a potential substitute for formalin with further assessment and improvement of current protocols.

## Availability and requirements

Project name: fqtrim

Project home page: http://ccb.jhu.edu/software/fqtrim/index.shtml

Operating system(s): Linux

Programming language: C++

License: fqtrim is free, open source software released under an Artistic License.

Any restrictions to use by non-academics: None.

## Additional files

The data sets supporting the results of this article are included within the article and its additional files.

10.1186/s13104-015-1725-4 Table of actin PCR primers and probes.
10.1186/s13104-015-1725-4 Table of NGS mean coverage by gene.

## Abbreviations

SCP: Streck cell preservative
NGS: Next generation sequencing
NBF: 10 % neutral buffered formalin
FISH: Fluorescence in situ hybridization
ddPCR: Droplet digital PCR

## References

[1] Fox CH, Johnson FB, Whiting J, Roller PP (1985). Formalin fixation. J Histochem Cytochem.

[2] Costa S, Coelho P, Costa C, Silva S, Mayan O, Santos LS (2008). Genotoxic damage in pathology anatomy laboratory workers exposed to formalin. Toxicol.

[3] Werner M, Chott A, Fabiano A, Battifora H (2000). Effect of formalin tissue fixation and processing on immunohistochemistry. Am J Surg Pathol..

[4] Srinivasan M, Sedmak D, Jewell S (2002). Effect of fixatives and tissue processing on the content and integrity of nucleic acids. Am J Pathol.

[5] Kailasam S, Rogers KR (2007). A fluorescence-based screening assay for DNA damage induced by genotoxic industrial chemicals. Chemosphere.

[6] Williams C, Ponten F, Moberg C, Söderkvist P, Uhlen M (1999). A high frequency of sequence alterations is due to formalin fixation of archival specimens. Am J Pathol.

[7] Coombs NJ, Gough AC, Primrose JN (1999). Optimization of DNA and RNA extraction from archival formalin-fixed tissues. Nucleic Acid Res.

[8] Do H, Wong SQ, Li J, Dobrovic A (2013). Reducing sequence artifacts in amplicon-based massively parallel sequencing of formalin-fixed paraffin-embedded DNA by enzymetic depletion of uracil-containing templates. Clin Chem.

[9] Gatta LB, Cadei M, Balzarini P, Castriciano S, Paroni R (2012). Application of alternative fixatives to formalin in diagnostic pathology. Eur J Histochem.

[10] Das K, Dumais J, Basiaga S, Krzyzanowski GD (2013). Carbon-13 nuclear magnetic resonance analysis of formalin free preservatives. Acta Histochem.

[11] Saxton JM, Pockley AG (1998). Effect of ex vivo storage on human peripheral blood neutrophil expression of CD11b and the stabilizing effects of Cyto-Chex™. J Immunol Methods.

[12] Hindson BJ, Ness KD, Masquelier DA, Belgrader P, Heredia NJ, Makarewicz AJ (2011). High-throughput droplet digital PCR system for absolute quantification of DNA copy number. Anal Chem.

[13] Wolff AC, Hammond ME, Hicks DG, Dowsett M, McShane LM (2013). Recommendations for human epidermal growth factor receptor 2 testing in breast cancer: American society of clinical oncology/college of American pathologists clinical practice guideline update. J Clin Oncol.

[14] Hui P (2014). Next generation sequencing: chemistry, technology and applications. Top Curr Chem.

[15] Heredia NJ, Belgrader P, Wang S, Koehler R, Regan J (2013). Droplet digital™ PCR quantitation of HER2 expression in FFPE breast cancer samples. Methods.

[16] Maes RK, Langohr IM, Wise AG, Smedley RC, Thaiwong T, Kiupel M (2014). Beyond H&E: integration of nucleic acid-based analyses into diagnostic pathology. Vet Pathol.

[17] Serth J, Kuczyk MA, Paeslack U, Lichtinghagen R, Jonas U (2000). Quantitation of DNA extracted after micropreparation of cells from frozen and formalin-fixed tissue sections. Am J Pathol.

[18] Shigeki N, Mohammad G, Yuichi S (2005). Reversing the effects of formalin fixation with citraconic anhydride and heat: a universal antigen retrieval method. J Histochem Cytochem.

[19] Ahn SJ, Costa J, Emanuel JR (1996). PicoGreen quantitation of DNA: effective evaluation of samples pre- or post-PCR. Nucleic Acids Res.

[20] Das K, Fernando MR, Basiaga S, Wigginton SM, Williams T (2014). Effects of a novel cell stabilizing reagent on DNA amplification by PCR as compared to traditional stabilizing reagents. Acta Histochem.

[21] Pat SK, Chowdhury A, Sivaamnuaiphorn S, Beryt M, Pegram M (2005). Characterization of HER2 overexpressing breast cell lines selected for long-term culture with trastuzumab. Proc Amer Assoc Cancer Res.

[22] Bass BP, Engel KB, Greytak SR, Moore HM (2014). A review of preanalytical factors affecting molecular, protein, and morphological analysis of formalin-fixed, paraffin-embedded (FFPE) tissue: how well do you know your FFPE specimen?. Arch Pathol Lab Med.

[23] Vincek V, Nassiri M, Nadji M, Morales AR (2003). A tissue fixative that protects macromolecules (DNA, RNA, and protein) and histomorphology in clinical samples. Lab Invest.

[24] Howat WJ, Wilson BA (2014). Tissue fixation and the effect of molecular fixatives on downstream staining procedures. Methods.

