# Supplementary material for: A formalin-free method for stabilizing cells for nucleic acid amplification, hybridization and next-generation sequencing
Source: BMC Res Notes. 2015 Dec 9;8:755. doi: 10.1186/s13104-015-1725-4 (PMC4673747; doi:10.1186/s13104-015-1725-4)
Supplement: Supplementary file 1 — 10.1186/s13104-015-1725-4 Table of actin PCR primers and probes. [file 13104_2015_1725_MOESM1_ESM.docx]

Forward Primer Reverse Primer Probe Amplicon Length

5’-GGT GTT TGT CTC TCT GAC TAG G-3’ 5’-CAC ACG AGC CAG TGT TAG TA-3’ 5HEX-TGT CTG AGA CAG TGT TGT GGG TGT_BHQ 69 bp

5’-ACT CTG CAG GTT CTA TTT GCT-3’ 5’-ACT CCA AGG CCG CTT TAC-3’ 6FAM-ACC CAC AAC ACT GTC TCA GAC ACC_BHQ 152 bp

5′-CCG CTA CCT CTT CTG GTG-3′ 5′-GAT GCA CCA TGT CAC ACT-3′ 6FAM-CCT CCC TCC TTC CTG GCC TC_BHQ 420 bp

**Table S1: β-actin Primers and Probes for each Amplicon Size by ddPCR**
