# Supplementary material for: A formalin-free method for stabilizing cells for nucleic acid amplification, hybridization and next-generation sequencing
Source: BMC Res Notes. 2015 Dec 9;8:755. doi: 10.1186/s13104-015-1725-4 (PMC4673747; doi:10.1186/s13104-015-1725-4)
Supplement: Supplementary file 2 — 10.1186/s13104-015-1725-4 Table of NGS mean coverage by gene. [file 13104_2015_1725_MOESM2_ESM.docx]

| Gene Name  **Table 2S: NGS Mean Depth-of-Coverage by Gene** | Size_of_Gene | mean-depth | | p value |
| --- | --- | --- | --- | --- |
|  |  | Frozen control | SCP |  |
| *SDHB* | 843 | 322.7 | 469.7 | *0.15* |
| *MUTYH* | 1650 | 512.7 | 757.3 | *0.12* |
| *SDHC* | 510 | 627.1 | 899.2 | *0.19* |
| *CDC73* | 1596 | 597.1 | 800.0 | *0.29* |
| *FH* | 1533 | 544.9 | 801.7 | *0.20* |
| *ALK* | 4863 | 534.2 | 803.0 | *0.12* |
| *EPCAM* | 945 | 372.0 | 503.0 | *0.25* |
| *MSH2* | 2805 | 464.7 | 647.2 | *0.25* |
| *MSH6* | 4083 | 474.2 | 719.0 | *0.17* |
| *FANCL* | 1143 | 413.2 | 559.9 | *0.29* |
| *TMEM127* | 717 | 184.1 | 280.7 | *0.09* |
| *ERCC3* | 2349 | 529.0 | 804.0 | *0.14* |
| *PMS1* | 2799 | 355.4 | 502.8 | *0.28* |
| *DIS3L2* | 2658 | 352.1 | 509.5 | *0.12* |
| *FANCD2* | 4416 | 385.4 | 542.4 | *0.20* |
| *VHL* | 642 | 429.9 | 653.6 | *0.09* |
| *XPC* | 2823 | 849.7 | 1228.8 | *0.15* |
| *MLH1* | 2271 | 779.1 | 1132.5 | *0.19* |
| *BAP1* | 2190 | 150.4 | 217.6 | *0.13* |
| *GATA2* | 1443 | 200.2 | 303.0 | *0.07* |
| *PHOX2B* | 945 | 259.0 | 387.9 | *0.09* |
| *KIT* | 2931 | 524.7 | 769.5 | *0.18* |
| *APC* | 8532 | 383.1 | 594.9 | *0.17* |
| *NSD1* | 8091 | 455.7 | 690.0 | *0.16* |
| *FANCE* | 1611 | 371.8 | 542.0 | *0.15* |
| *PMS2* | 2589 | 906.9 | 1312.0 | *0.19* |
| *EGFR* | 3633 | 771.5 | 1123.4 | *0.14* |
| *SBDS* | 753 | 875.9 | 1274.2 | *0.21* |
| *MET* | 4227 | 731.0 | 1099.5 | *0.17* |
| *EZH2* | 2256 | 435.6 | 609.1 | *0.21* |
| *WRN* | 4299 | 368.2 | 505.7 | *0.27* |
| *NBN* | 2265 | 2466.7 | 3370.4 | *0.23* |
| *EXT1* | 2241 | 1765.5 | 2548.4 | *0.16* |
| *RECQL4* | 3628 | 626.5 | 882.1 | *0.11* |
| *CDKN2A* | 664 | 331.9 | 484.3 | *0.10* |
| *FANCG* | 1869 | 342.1 | 526.0 | *0.12* |
| *FANCC* | 1677 | 557.1 | 802.9 | *0.19* |
| *PTCH1* | 4341 | 516.6 | 769.7 | *0.14* |
| *XPA* | 822 | 570.6 | 791.9 | *0.25* |
| *TSC1* | 3495 | 548.3 | 798.1 | *0.17* |
| *RET* | 3345 | 541.4 | 802.0 | *0.11* |
| *PRF1* | 1668 | 773.2 | 1159.7 | *0.12* |
| *BMPR1A* | 1599 | 363.7 | 493.2 | *0.22* |
| *PTEN* | 1212 | 298.6 | 391.6 | *0.33* |
| *SUFU* | 1455 | 281.4 | 421.5 | *0.12* |
| *HRAS* | 570 | 480.9 | 689.8 | *0.12* |
| *CDKN1C* | 951 | 487.1 | 664.1 | *0.07* |
| *FANCF* | 1125 | 544.0 | 827.2 | *0.13* |
| *WT1* | 1554 | 524.9 | 770.2 | *0.11* |
| *EXT2* | 2256 | 469.8 | 689.4 | *0.17* |
| *DDB2* | 1284 | 563.6 | 842.8 | *0.16* |
| *SDHAF2* | 501 | 482.8 | 709.7 | *0.15* |
| *MEN1* | 1848 | 522.0 | 761.2 | *0.12* |
| *AIP* | 1227 | 748.5 | 1079.8 | *0.14* |
| *CEP57* | 1503 | 516.3 | 752.6 | *0.21* |
| *ATM* | 3409 | 454.2 | 637.4 | *0.24* |
| *SDHD* | 480 | 355.2 | 542.9 | *0.15* |
| *CDK4* | 912 | 487.1 | 716.4 | *0.16* |
| *HNF1A* | 1896 | 264.4 | 390.6 | *0.08* |
| *BRCA2* | 10257 | 430.6 | 622.8 | *0.24* |
| *RB1* | 2787 | 447.3 | 588.7 | *0.31* |
| *ERCC5* | 3561 | 754.3 | 1127.2 | *0.18* |
| *FANCM* | 6147 | 460.1 | 641.2 | *0.28* |
| *MAX* | 483 | 483.3 | 745.4 | *0.12* |
| *DICER1* | 5769 | 427.2 | 635.9 | *0.20* |
| *BUB1B* | 3153 | 459.8 | 651.3 | *0.23* |
| *FANCI* | 3987 | 409.4 | 598.9 | *0.20* |
| *BLM* | 4254 | 426.8 | 621.4 | *0.23* |
| *TSC2* | 5424 | 562.1 | 814.0 | *0.11* |
| *SLX4* | 5505 | 647.7 | 968.7 | *0.14* |
| *ERCC4* | 2751 | 581.5 | 863.7 | *0.19* |
| *PALB2* | 3561 | 482.5 | 733.1 | *0.21* |
| *CYLD* | 2871 | 468.8 | 661.6 | *0.22* |
| *CDH1* | 2649 | 86.9 | 127.5 | *0.15* |
| *FANCA* | 4368 | 288.5 | 431.3 | *0.14* |
| *TP53* | 1182 | 333.8 | 491.6 | *0.14* |
| *FLCN* | 1740 | 431.7 | 636.4 | *0.14* |
| *NF1* | 8520 | 257.3 | 370.7 | *0.21* |
| *RAD51D* | 987 | 231.5 | 355.8 | *0.16* |
| *BRCA1* | 5592 | 254.2 | 376.8 | *0.24* |
| *RAD51C* | 1131 | 252.6 | 351.2 | *0.30* |
|  |  |  |  |  |
| *BRIP1* | 3750 | 251.0 | 351.7 | *0.28* |
| *PRKAR1A* | 1146 | 803.8 | 1154.1 | *0.16* |
| *RHBDF2* | 2571 | 417.2 | 588.1 | *0.11* |
| *SMAD4* | 1659 | 231.9 | 344.6 | *0.19* |
| *STK11* | 1302 | 415.8 | 607.5 | *0.09* |
| *CEBPA* | 1077 | 412.6 | 586.5 | *0.05* |
| *ERCC2* | 2283 | 459.5 | 691.1 | *0.10* |
| *RUNX1* | 1443 | 247.2 | 366.3 | *0.12* |
| *SMARCB1* | 1158 | 358.9 | 572.4 | *0.10* |
| *CHEK2* | 1632 | 382.2 | 542.7 | *0.19* |
| *NF2* | 1788 | 469.0 | 700.0 | *0.13* |
| *FANCB* | 2580 | 511.9 | 704.3 | *0.30* |
| *GPC3* | 1743 | 325.9 | 505.9 | *0.19* |
